# Social Distancing and Lockdown – An Introvert’s Paradise? An Empirical Investigation on the Association Between Introversion and the Psychological Impact of COVID19-Related Circumstantial Changes

**DOI:** 10.3389/fpsyg.2020.561609

**Published:** 2020-09-17

**Authors:** Maryann Wei

**Affiliations:** School of Psychology, University of Wollongong, Wollongong, NSW, Australia

**Keywords:** COVID19, introversion, extraversion, personality, loneliness, anxiety, depression, cognitive failures

## Abstract

The coronavirus disease 2019 (COVID19) pandemic has effected the implementation of social distancing and lockdown measures across the globe, and the psychological impact of associated life changes is experienced more severely by some individuals than others. Anecdotal evidence points to a common belief among the general public that introverts are faring better than their extraverted counterparts to this end. However, the claim lacks empirical research, and seems counterintuitive when the broader literature on the association between introversion and mental health is considered. The current study investigated whether the psychological impact of COVID19-related circumstantial changes was moderated by introversion, based on outcome measures across psychosocial, cognitive, and affective domains. The role of several demographic factors in determining COVID19-related mental health symptoms was also examined. One hundred and fourteen individuals (64 USA residents) completed measures of introversion, and reported on the extent to which they experienced loneliness, anxiety, depression and cognitive impairments as a function of COVID19-related circumstantial changes. Results showed that introversion predicted more severe loneliness, anxiety, and depression experienced as a function of COVID19-related circumstantial changes, but not cognitive impairments. Among the range of demographic factors examined (age, gender, living condition, recent unemployment), living with others (vs. living alone) predicted more severe COVID19-related mental health symptoms. However, these effects were only observed on outcome measures pertaining to anxiety and cognitive impairments, but not loneliness and depression. Current findings have implications for both consumers and disseminators of information on popular internet hubs. Current findings also highlight the possibility that living with others (close human affiliation) may have protective and detrimental effects on different domains of mental health during the COVID19 pandemic.

## Introduction

The introversion-extraversion dimension is central to leading trait theories of human personality in psychology (e.g., Myers, 1962; Cattell, 1965; Eysenck, 1967; Hathaway, 1982; McCrae and Costa, 1999, although exact conceptualisations vary between theories), and the construct is ubiquitous in both academic and popular literature. Commonly described in dichotomic terms, introverts and extraverts are often differentiated by the sources they draw energy from (internal vs. external, respectively). Adjectives traditionally associated with introversion include “inhibited,” “reserved,” and “undemonstrative,” while those associated with extraversion include “outgoing,” “friendly,” and “enthusiastic” (Eysenck, 1991).

The coronavirus disease 2019 (COVID19) pandemic has effected the implementation of social distancing and lockdown measures across the globe, and the psychological impact of associated life changes is experienced more severely by some individuals than others (Williams et al., 2020). Anecdotal evidence points to a common belief among the general public that introverts are faring better than their extraverted counterparts to this end. For example, a “How to Survive Social Distancing as an Extravert” guide on a popular psychology website begins with the following statements: “*For introverts, being stuck at home without social interaction for long periods of time really isn’t the worst thing at all. They are accustomed to this time spent alone and feel energized and recharged by it. When it comes to extroverts, the idea of social distancing can feel like somewhat of a death sentence”* (Personality Growth, 2020). In articles published on other widely-frequented non-psychology websites, introversion has been championed as an asset for thriving in COVID19-related social isolation (e.g., Bloomberg, 2020; Reuters, 2020; The Conversation, 2020). Such beliefs are exemplified in the influx of user-generated pictorial content (more colloquially known as “memes”) across the internet with similar sentiments (see Supplementary Material for exemplars (Data Sheet 1)).

On that grounds that introverts prefer less stimulating environments (Myers, 1962; Cattell, 1965; Eysenck, 1967; Hathaway, 1982; McCrae and Costa, 1999), the assumption that introverts experience the psychological impact of COVID19-related circumstantial changes less severely than extraverts seems plausible. However, the claim lacks empirical research, and there are several lines of work in light of which the claim appears counterintuitive. First, introversion has been linked to personality traits associated with the tendency to experience more intense emotions and more difficulties in regulating these emotions, namely the “feeling” dimension of the Myer-Briggs Type Indicator (Janowsky, 2001) and neuroticism, respectively (Janowsky, 2001; Jylha et al., 2009; Fadda and Scalas, 2016). Additionally, and possibly resultantly, introversion has also been associated with more psychological problems in general (Janowsky, 2001; Jylha et al., 2009; Fadda and Scalas, 2016), and adjustment problems in particular. Specifically, studies have demonstrated that introverts struggle more than extraverts in adjusting to life events which entail changes in day-to-day life, including shifts between educational institutes (Bauer and Liang, 2003; Davidson et al., 2015), job relocation (Pinder, 1977), and retirement (Löckenhoff et al., 2009; Robinson et al., 2010). Although increased amounts of time alone should in theory be welcome by introverts, these findings raise questions on whether introverts necessarily have an advantage over their extraverted counterparts in adapting to COVID19-related circumstantial changes. Additionally, the psychological impact of COVID19-related circumstantial changes (and mental health in general) has psychosocial, cognitive, and affective aspects, which in turn represent functional domains which may be differentially moderated by personality traits (Segel-Karpas and Lachman, 2018).

The primary aim of this study was to examine whether the psychological impact of COVID19-related circumstantial changes is moderated by introversion, based on outcome measures across psychosocial, cognitive, and affective domains. A second aim was to examine the unique role of several other demographic factors (which were also considered as control variables in fulfilling the primary aim) in determining COVID19-related mental health symptoms.

## Materials and Methods

### Participants

Between late April and early May 2020^1^, a call for participants for a study on the psychological impact of COVID19 was placed on the sub-reddit r/SampleSize, an online platform designated to connect researchers and research volunteers. Based on previous research (Shatz, 2016; Jamnik and Lane, 2017) and the current author’s own experience, recruitment using this platform reliably produces quality data from adult individuals dominantly residing in the United States of America (USA). The latter demographic profile seems appropriate for the current research, given the high incidence of COVID19 in the USA and the strictness of lockdown/social distancing measures which ensued.

One hundred and fourteen individuals responded to the call for participants (Mean age = 30.52, *SD* = 10.02; 85 Female). Sixty two respondents were located in the USA. The other 52 respondents were distributed across the following countries, including United Kingdom (*N* = 14), Canada (*N* = 5), Australia (*N* = 4), Germany (*N* = 3). USA and non-USA residents were compared on all outcome variables (described below) to identify cases where the current sample could not be considered as a whole.

### Measures

#### Predictor Variables

Introversion-Extraversion was measured as a continuous dimension using the Introversion Scale developed by Richmond and McCroskey (1998)^2^. This scale was developed based on the Extraversion subscale in the Eysenck Personality Questionnaire (Eysenck et al., 1985). To illustrate, items such as “*Can you usually let yourself go and enjoy yourself at a lively party?”* and “*Do you tend to keep in the background on social occasions?”* in the Eysenck Personality Questionnaire have counterparts in Richmond and McCroskey’s Introversion Scale in *“Can you usually let yourself go and have a good time at a party?”* and *“Are you inclined to keep in the background on social occasions?,”* respectively^3^. The Introversion Scale consists of 18 such statements. Respondents indicate whether each statement applied to them on a 5-point scale ranging from Strongly Disagree (1) to Strongly Agree (5). Six statements serve as distractors and are not scored. Alpha reliability estimates were above.80 in the initial validation study by Richmond and McCroskey (1998), and closely matched in the current study (Cronbach’s alpha = 0.77). Scores range between 12 and 60, with higher scores indicating higher introversion, and lower scores indicating lower introversion (i.e., higher extraversion).

The following demographics were measured as predictor variables of interest: Age, Gender, Living condition (Alone/With others), Recent unemployment due to COVID19 (No/Yes).

#### Outcome Variables

The psychological impact of COVID19-related circumstantial changes was measured with a battery of established questionnaires, with instructions modified to elicit mental health ratings directly associated with the implementation of social distancing and lockdown measures. That is, instead of reporting on mental health symptoms based on a given retrospective timeframe, participants were asked to provide ratings as a function of COVID19-related circumstantial changes. The exact phrase of instructions participants received is detailed in context below. Functional aspects in the psychosocial, cognitive, and affective domains were measured, with the affective domain further broken down into depressive and anxious sub-domains.

##### Psychosocial domain

Participants completed the DeJong Gierveld Loneliness Scale (De Jong Gierveld and Van Tilburg, 2006) to provide an indicator of loneliness as a function of COVID19-related circumstantial changes. Participants responded “No”, “More or less,” or “Yes” to six statements, headed by the question “How true are these statements for you, following the implementation of COVID19 social distancing and lockdown measures?” On negatively-worded statements (e.g., “*I miss having people around me*”), “More or less” and “Yes” responses are scored as 1 while “No” responses are scored as 0. On positively-worded statements (e.g., “*There are enough people I can trust completely*”), “More or less” and “No” responses are scored as 1 while “Yes” responses are scored as 0. Scores are summed across 6 items (range 0–6), where higher scores indicate higher loneliness. Reliability and validity of the scale has been demonstrated across the lifespan (De Jong Gierveld and Van Tilburg, 2010). Cronbach’s alpha for the De Jong Gierveld Loneliness Scale as presented in the current sample was slightly below the conventional acceptable benchmark of 0.70 at 0.64; however, Cronbach’s alphas bordering on 0.70 were observed in the initial validation study (De Jong Gierveld and Van Tilburg, 2006), so that internal consistency estimates in a slightly lower tier are likely normative given the few number of items in the scale (Tavakol and Dennick, 2011).

##### Cognitive domain

Cognitive impairments associated with COVID19-related circumstantial changes were assessed with the Cognitive Failures Questionnaire (CFQ; (Broadbent et al., 1982), a 25-item inventory of self-reported day-to-day slips and errors in cognition. Instructions were phrased as follows: “The following questions are about minor mistakes which everyone makes from time to time, but some of which happen more often than others. We would like to know how often these things have happened to you, following the implementation of COVID19 social distancing and lockdown measures.” Respondents indicated on a scale ranging from 0 (never) to 4 (very often) how often they experience certain incidents (e.g., “*Do you find you forget what you came to the shops to buy?*”). Scores (range 0–100) are summed across all items, where higher scores indicate more extreme cognitive impairments. In the initial pilot study, internal consistency of 0.89 was demonstrated (Broadbent et al., 1982). Cronbach’s alpha for the CFQ as presented in the current sample was 0.95.

##### Affective domain

***Depression***. Depressive symptoms associated with COVID19-related circumstantial changes were assessed with the Patient Health Questionnaire 9 [PHQ-9; (Kroenke and Spitzer, 2002)]. Instructions were phrased as follows: “To what extent (frequency) have you experienced these symptoms, following the implementation of COVID19 social distancing and lockdown measures?” Participants report the frequency with which they experience nine depressive symptoms on a 4-point scale ranging from “Not at all” (0) to “Nearly everyday” (3) (e.g., *Little interest or pleasure in doing things*). Scores are summed across the nine items (range 0–27), where higher scores indicate higher depression severity. The PHQ-9 has been validated not only as a useful tool to recognize clinical depression but also subthreshold depressive symptoms in the general population (Martin et al., 2006). Cronbach’s alpha for the PHQ-9 as presented in the current sample was 0.90.

***Anxiety***. Anxious symptoms associated with COVID19-related circumstantial changes were assessed with the Generalized Anxiety Disorder Screener (GAD-7; (Spitzer et al., 2006). The response format for the GAD-7 is identical to that of the PHQ-9. Instructions for the GAD-7 were also identical to that which were presently used for the PHQ-9. Scores are summed across seven items (i.e., seven symptoms of anxiety; e.g., *Not being able to stop or control worrying*), where higher scores indicate higher anxiety severity (range 0–21). Similar to the PHQ-9, the GAD-7 has been demonstrated as a reliable and valid measure of anxiety in the general population (Löwe et al., 2008). Cronbach’s alpha for the GAD-7 as presented in the current sample was 0.92.

### Data Analyses

All analyses described as follows (including the generation of descriptives) were processed with SPSS (Version 25). To evaluate whether introversion moderates the psychological impact of COVID19-related circumstantial changes, scores on the Introversion Scale were used to predict each outcome variable listed above. Hierarchical regression analyses were used, with demographic factors entered in the first step as control variables. The predictive value of each demographic factor across the range of outcome variables was also of research interest (pertaining to the second aim). Where significant differences were observed on outcome variables between USA and non-USA residents, regression analyses were performed separately for the two groups. For comprehensiveness, the two groups were also compared on all predictor variables.

## Results

Table 1 gives means and correlations between all study variables for the full sample. USA and non-USA residents did not differ on any of the predictor variables (age, gender, living condition, recent unemployment, and introversion). However, pertaining to outcome variables, USA and non-USA residents differed on the psychosocial domain. Specifically, USA residents reported experiencing higher loneliness as a function of COVID19-related circumstantial changes compared to non-USA residents (*M* = 4.00 vs. *M* = 3.25 on the DeJong Gierveld Loneliness Scale, respectively), *t*(112) = 2.39, *p* = 0.018. Thus, regression analyses predicting loneliness were performed separately for USA and non-USA residents.

**TABLE 1:** Means and correlations between all study variables.

	**1. Age**	**2. Gender (0 = Female)**	**3. Living condition (0 = Living alone)**	**4. Recent unemployment (0 = No)**	**5. Introversion (Introversion Scale)**	**6. Loneliness (DeJong Gierveld Loneliness Scale)**	**7. Cognitive failures (CFQ)**	**8. Depression (PHQ-9)**	**9. Anxiety (GAD-7)**
9.	–0.021	0.049	0.173	–0.004	0.177	0.395**	0.518**	0.776**	–
8.	–0.143	–0.035	0.161	0.049	0.190*	0.516**	0.629**	–	–
7.	–0.027	–0.028	0.212*	0.015	0.019	0.292**	–	–	–
6.	–0.010	0.027	–0.093	0.156	0.111	–	–	–	–
5.	0.010	–0.005	–0.061	0.060	–	–	–	–	–
4.	0.163	–0.156	–0.039	–	–	–	–	–	–
3.	−0.192*	–0.041	–	–	–	–	–	–	–
2.	–0.034	–	–	–	–	–	–	–	–
Mean [*SD*]	30.34 [10.26]	Female = 0 (*N* = 70); Male = 1 (*N* = 44)	Alone = 0 (*N* = 20); With Others = 1 (*N* = 94)	No = 0 (*N* = 84); Yes = 1 (*N* = 30)	39.73 [7.76]	3.66 [1.70]	36.40 [18.12]	10.33 [6.70]	8.21 [5.92]

* p < 0.05; ** p < 0.01.

Table 2 gives standardized β coefficients for predictor variables and associated model statistics in hierarchical regression analyses predicting the psychological impact of COVID19-related circumstantial changes, across psychosocial, cognitive, and affective domains. After controlling for age, gender, living condition and recent unemployment, higher introversion (higher Introversion Scale scores) uniquely predicted higher depression (PHQ-9) and anxiety (GAD-7) experienced as a function of COVID19-related circumstantial changes, β = 0.196, *t* = 2.12, *p* = 0.036 and β = 0.188, *t* = 2.02, *p* = 0.046, respectively. Higher introversion also uniquely predicted loneliness (DeJong Gierveld Loneliness Scale) experienced as a function of COVID19-related circumstantial changes after controlling for demographic factors, although this effect was unique to USA residents (β = 0.286, *t* = 2.27, *p* = 0.027). Introversion did not predict cognitive impairments (CFQ) related to COVID19 circumstantial changes after controlling for demographic variables (β = 0.031, *t* = 0.324, *p* = 0.747).

**TABLE 2:** Standardized β coefficients for predictor variables (and associated model statistics) in hierarchical regression analyses predicting the psychological impact of COVID19-related circumstantial changes, across psychosocial, cognitive, and affective domains.

	**Loneliness (USA residents; *N* = 62)**	**Loneliness (Non-USA residents; *N* = 52)**	**Cognitive failures (Full sample; *N* = 112)**	**Depression (Full sample; *N* = 112)**	**Anxiety (Full sample; *N* = 112)**
**Step 1**					
**Predictor**					
Age	–0.182	0.039	0.010	–0.129	0.014
Gender	–0.028	0.059	–0.016	–0.023	0.058
Living condition	–0.143	–0.164	0.214*	0.138	0.178
Recent unemployment	–0.016	0.338*	0.019	0.071	0.009
**Model statistics**					
*F*	0.670	10.73	10.31	10.28	0.940
*R*^2^	0.045	0.128	0.046	0.045	0.033

**Step 2**					
**Predictor**					
Age	–0.185	0.039	0.011	–0.127	0.016
Gender	–0.052	0.056	–0.016	–0.023	0.058
Living condition	–0.165	–0.171	0.216*	0.150	0.190*
Recent unemployment	–0.019	0.340*	0.017	0.060	–0.002
Introversion	0.286*	–0.039	0.031	0.196*	0.188*
**Model statistics**					
*F*	10.60	10.37	10.06	10.96	0.159
*R*^2^	0.125	0.130	0.047	0.083	0.069
*ΔR*^2^	0.080*	0.001	0.001	0.038*	0.035*

* *p* < 0.05.

In a model including introversion, recent unemployment predicted higher loneliness experienced as a function of COVID19-related circumstantial changes only for non-USA residents (β = 0.340, *t* = 2.38, *p* = 0.022). Interestingly, after including introversion in the model, living with others (vs. alone) was associated with more severe cognitive impairments and anxiety experienced as a function of COVID19-related circumstantial changes (β = 0.216, *t* = 2.25, *p* = 0.027 and β = 0.190, *t* = 2.00, *p* = 0.048, respectively). It is worth noting that the living condition did not have predictive value for loneliness and depressive symptoms experienced as a function of COVID19-related circumstantial changes (see Table 2).

## Discussion

This study examined whether the psychological impact of COVID19-related circumstantial changes was moderated by introversion, based on outcome measures across psychosocial, cognitive, and affective domains. As a second aim of the current study, the role of several other demographic factors in determining COVID19-related mental health symptoms was also examined.

Overall, higher introversion (i.e., lower extraversion) was associated with higher loneliness, depression and anxiety experienced as a function of COVID19-related circumstantial changes. The finding that introverts experience the psychosocial and affective impact of social distancing and lockdown measures more severely than their extraverted counterparts converges and deviates from previous literature in several ways. First, the finding is in line with previous studies demonstrating that introversion is associated with more psychological problems in general (Janowsky, 2001; Jylha et al., 2009; Fadda and Scalas, 2016), and adjustment problems specifically (Pinder, 1977; Bauer and Liang, 2003; Löckenhoff et al., 2009; Robinson et al., 2010; Davidson et al., 2015), However, this finding appears to be in disagreement with the notion that introversion is associated with a preference for less stimulating environments (Myers, 1962; Cattell, 1965; Eysenck, 1967; Hathaway, 1982; McCrae and Costa, 1999) such as that created in everyday life following the implementation of social distancing and lockdown measures. In turn, this assumption has fuelled the lay belief that introverts are coping better during the COVID19 pandemic compared to extraverts (detailed in section “Introduction”).

Current findings may be best understood without considering the two lines of thought as mutually exclusive. Introversion has been linked to decreased help-seeking behavior (Swickert et al., 2002; Atik and Yalçin, 2011; Kakhnovets, 2011), which may in part explain higher psychological problems among introverts at baseline (Janowsky, 2001; Jylha et al., 2009; Fadda and Scalas, 2016). When experiencing negative emotions, introverts are similarly more likely turn inwardly to cope (Shapiro and Alexander, 1975). While introspective behaviors can facilitate emotional self-regulation, such habits can also function as a double-edged sword in perpetuating internalization (Bowker and Rubin, 2009), rumination (Verhaeghen et al., 2005; Cohen and Ferrari, 2010), and worry (Philippi and Koenigs, 2014) – key cognitive underpinnings of loneliness, depression, and anxiety, respectively (Beck, 2008; Newman et al., 2013; Ypsilanti, 2018). Such an account of why individuals higher on introversion might experience the psychosocial and affective impact of COVID19-related circumstantial changes more severely is corroborated by other aspects of present findings. Specifically, cognitive impairments experienced as a function of social distancing and lockdown measures were not moderated by introversion, suggesting that cognitive function and activity remains intact across the introversion-extraversion dimension through COVID19-related circumstantial changes. Current findings are in keeping with previous research demonstrating that functional domains of mental health are differentially moderated by personality traits (Segel-Karpas and Lachman, 2018), and highlight the particular relevance of evaluating domain-specific effects in research on the association between introversion and mental health. Crucially, these findings have implications for both consumers and disseminators of information on popular internet hubs – specifically, to keep in view that the notion of introverts thriving under lockdown and social distancing conditions may not necessarily be empirically supported. Mental health professionals dealing with COVID19-related psychological issues should also be aware that introverts may risk being erroneously left out of the mental health system.

One aspect of present observations is worth noting before proceeding to discuss findings pertaining to the second aim of the current study. Namely, the psychosocial impact of COVID19-related circumstantial changes (loneliness) was predicted by both introversion and demographic factors (specifically, recent unemployment), but these effects were unique to USA and non-USA residents, respectively. There are several possible explanations for this observation including: (1) predictors of COVID19-related loneliness differ qualitatively at different levels of loneliness severity, given USA residents reported higher loneliness as a function of COVID19-related circumstantial changes in the current sample, and (2) the psychosocial impact of COVID19-related circumstantial changes is predicted by qualitatively different factors in different cultures. While not within the scope of the present study’s aims, these speculations represent testable hypotheses which may be of interest in future research.

Besides recent unemployment, other demographic predictors examined were age, gender, and living condition. Only living condition made a unique contribution to COVID19-related mental health symptoms after accounting for introversion. Specifically, living with others (vs. living alone) was associated with experiencing more cognitive impairments and anxiety as a function of COVID19-related circumstantial changes. Adjacently, it was observed that COVID19-related loneliness and depressive symptoms were not predicted by living condition. Interpreted together, it is possible that close human affiliation serves as a protective buffer against social disconnectedness and low mood during the COVID19 pandemic, but works in the opposite direction for clarity of thought and keeping calm. Further information on household dynamics would have helped in the development of this speculation, but was not obtained in the present study.

Other limitations of the current study include its cross-sectional nature, so that pre-COVID19 mental health issues may have been conflated with COVID19-related mental health symptoms as presently assessed. On a related note, the current study assumes that presently used outcome measures (worded with reference to COVID19 social distancing and lockdown measures) captured psychological health as shaped specifically by social orders placed as preventative measures against COVID19. However, responses on these measures may also reflect psychological health as impacted by the global-scale pandemic more generally, so that responses may not be tied solely to increased amounts of solitary time. Further, demographic variables were considered only in broad strokes in the present study. Accounting for a wider range of demographic variables, including but not limited to income, would allow for a clearer picture of the association between introversion and the psychological impact of COVID19-related circumstantial changes to be drawn. Next, the participant count in each non-USA country was small in the present sample, so that non-USA respondents had to be collapsed in a single “non-USA residents” group. Although the COVID19 outbreak is considered a global pandemic, there may still be subtle differences in the COVID19 impact between countries. More targeted and selective recruitment according to location/residence should be considered in future research. Finally, given present interests in multiple outcome variables, the current study would have benefited in terms of statistical power from a larger sample size.

## Data Availability Statement

The datasets presented in this study can be found in online repositories. The names of the repository/repositories and accession number(s) can be found in the article/ Supplementary Material.

## Ethics Statement

Ethical review and approval was not required for the study on human participants in accordance with the local legislation and institutional requirements. The patients/participants provided their written informed consent to participate in this study.

## Author Contributions

The author confirms being the sole contributor of this work and has approved it for publication.

## Conflict of Interest

The authors declare that the research was conducted in the absence of any commercial or financial relationships that could be construed as a potential conflict of interest.

## Footnotes

1 Within this timeframe, social distancing and lockdown measures were in full swing in most parts of the world.

2 This scale is kindly made available by McCroskey at http://www.jamescmccroskey.com/measures/introversion.htm.

3 The Introversion Scale (Richmond and McCroskey, 1998) was presently selected over the Eysenck Personality Questionnaire (and other more commonly used personality test batteries) to favor survey succinctness and participant retention. The option of selectively presenting only introversion-related items from larger personality test batteries was avoided as this would require altering the dispersion of items in the order they have been validated.
