# Supplementary material for: Social Distancing and Lockdown – An Introvert’s Paradise? An Empirical Investigation on the Association Between Introversion and the Psychological Impact of COVID19-Related Circumstantial Changes
Source: Front Psychol. 2020 Sep 17;11:561609. doi: 10.3389/fpsyg.2020.561609 (PMC7527530; doi:10.3389/fpsyg.2020.561609)
Supplement: Supplementary file 1 [file Data_Sheet_1.docx]

Supplementary Material


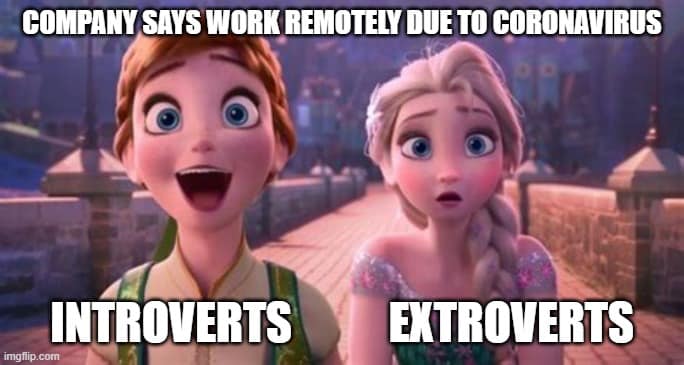


Source: Facebook – [Introvert Memes](https://www.facebook.com/introvertmemes/)


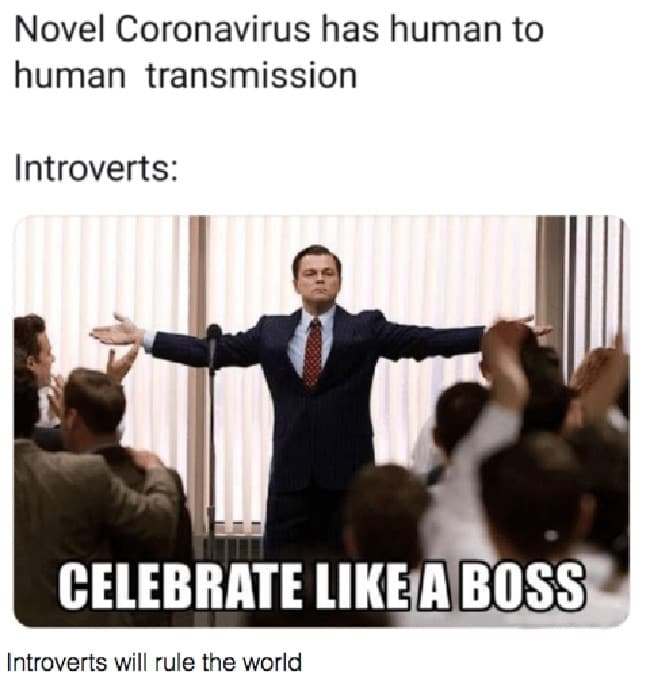


Source: [The Daily Telegraph](file:///E:\Forum%20Analysis\dailytelegraph.com.au\rendezview\hey-introverts-stop-loving-this-crisis-so-much\news-story\a519a8644d149222b0c22c97aa2c28c2)
